# Treatment of Manure and Digestate Liquid Fractions Using Membranes: Opportunities and Challenges

**DOI:** 10.3390/ijerph18063107

**Published:** 2021-03-17

**Authors:** Maria Salud Camilleri-Rumbau, Kelly Briceño, Lene Fjerbæk Søtoft, Knud Villy Christensen, Maria Cinta Roda-Serrat, Massimiliano Errico, Birgir Norddahl

**Affiliations:** 1 Department of Green Technology, Faculty of Engineering, University of Southern Denmark, Campusvej 55, 5230 Odense, Denmark; kellybm2004@gmail.com (K.B.); lfj@kbm.sdu.dk (L.F.S.); kvc@igt.sdu.dk (K.V.C.); mcs@igt.sdu.dk (M.C.R.-S.); maer@igt.sdu.dk (M.E.); bno@igt.sdu.dk (B.N.); 2 Aquaporin A/S, Nymøllevej 78, 2800 Kongens Lyngby, Denmark

**Keywords:** anaerobic digestion, digestate, liquid-solid separation, membrane separation, nutrient recovery, membrane fouling

## Abstract

Manure and digestate liquid fractions are nutrient-rich effluents that can be fractionated and concentrated using membranes. However, these membranes tend to foul due to organic matter, solids, colloids, and inorganic compounds including calcium, ammonium, sodium, sulfur, potassium, phosphorus, and magnesium contained in the feed. This review paper is intended as a theoretical and practical tool for the decision-making process during design of membrane-based systems aiming at processing manure liquid fractions. Firstly, this review paper gives an overview of the main physico-chemical characteristics of manure and digestates. Furthermore, solid-liquid separation technologies are described and the complexity of the physico-chemical variables affecting the separation process is discussed. The main factors influencing membrane fouling mechanisms, morphology and characteristics are described, as well as techniques covering membrane inspection and foulant analysis. Secondly, the effects of the feed characteristics, membrane operating conditions (pressure, cross-flow velocity, temperature), pH, flocculation-coagulation and membrane cleaning on fouling and membrane performance are presented. Finally, a summary of techniques for specific recovery of ammonia-nitrogen, phosphorus and removal of heavy metals for farm effluents is also presented.

## 1. Introduction

Animal manure was defined by Shobert and Maguire as the solid, semisolid, and liquid by-product generated by animals grown to produce meat, milk, eggs, and other agricultural products for human use and consumption [1]. Over a billion tonnes of manure are annually produced in the Unites States and 1.4 billion tonnes in Europe [2,3].

Animal manure represents a valuable fertilizer. It is estimated that globally livestock manure provided over 115 million tonnes of nitrogen (N) as input to agricultural soils in 2017 [4]. Nevertheless, this resource needs to be carefully managed to avoid ammonia emissions and nutrient losses to water recipients. In the last decades, changes in animal production have resulted in increased production of wastewater volumes, pollution of air, aquifers, surface waters and soil [5,6,7]. The intensive livestock production has led to challenges in the management, treatment and distribution of the manure nutrients, increasing difficulties in planning solution and investing in technologies to effectively valorize the manure produced. Manure is responsible for 7% of both agriculture CH_4_ and N_2_O emissions, being the second largest source of greenhouse gas emissions on a dairy farm [8]. It is estimated that methane emissions resulting from manure management have increased by 21% between 1990 and 2020, reaching 500 million tonnes of CO_2_ equivalents [9]. In this context, the development and implementation of technologies for manure valorization appear of paramount importance.

Anaerobic digestion, which is a common practice in some European regions with intensive farming, is an efficient biomass treatment to reduce greenhouse gas emissions and produce energy [10,11,12]. During anaerobic digestion, the organic matter is converted into biogas containing primarily methane (CH_4_) and carbon dioxide (CO_2_). The obtained digestate is rich in primary nutrients and can improve soil structure when applied in agriculture, by helping soil particles to bind together into aggregates [13]. This improves soil nutrient and water holding capacity preventing erosion [14]. Moreover, the use of efficient separation techniques for the digestate could be beneficial in the overall management of animal waste.

The separation of the digestate into a liquid and a solid fraction is recommended for reducing animal waste volumes and the costs associated to transportation. Solid-liquid separation is also beneficial for producing a concentrated and ready-to-use agricultural fertilizer [5,15,16]. The organic matter contained in the digestate solid fraction provides a readily available carbon source improving the biological, chemical and physical soil characteristics as a soil amendment [13,17,18]. However, solid-liquid separation does not guarantee high recovery of the nutrients still available in the obtained liquid fraction. Therefore, if a further post-treatment of the still diluted liquid fraction is required, membrane filtration offers an efficient technical solution able to re-distribute the unbalanced nutrient concentration on the resulting liquid fraction [19,20,21,22]. Membrane technologies, including microfiltration (MF), ultrafiltration (UF), nanofiltration (NF) and reverse osmosis (RO), are extensively used for water and wastewater treatment and have been used before for separation of animal waste sources. Alternative technologies such as forward osmosis (FO), electrodialysis (ED) and membrane distillation (MD) have also raised attention for recovery of nutrients from farm effluents [19,20,23,24,25,26,27,28,29].

However, membrane technologies have been used to a limited extent during separation of farm effluents. This limited application is mainly due to the challenges related in using membrane technologies. One of the main limitations is fouling, which leads to permeate flux decay and ultimately to the loss of membrane performance [30,31]. Despite membrane fouling being the main challenge during wastewater filtration, it can be controlled by establishing an efficient operation and membrane pretreatment. In this regard, Pontie et al. [32] suggested that using membrane systems for RO pretreatment in seawater feed is by far the best available technology due to ease of operation, low footprint and lower chemical usage compared to conventional pretreatment systems. During effluent nutrient recovery, MF and UF membranes perform as very efficient solid-liquid separators that can reject nutrients associated with particles such as phosphorus [33,34], whereas NF, RO, FO, MD and ED can be used for the separation and concentration of nitrogen compounds and potassium [20,28,29,34].

In this review, nutrient recovery from raw and digested manure using membranes is discussed, with focus on the state-of-the-art on membrane separation applied to raw manure and anerobic digestate liquid fractions. Factors affecting the membrane nutrient separation performances such as feed composition, membrane pretreatment and operation, membrane fouling, membrane cleaning strategies and membrane ageing are also described. This review guides the reader in understanding the application of membrane technology to farm effluents, considering opportunities and challenges.

## 2. Methodology

The methodology followed is a state-of-the-art review approach, with an angle towards a critical review on manure and digestate post-treatment using membranes [35,36]. The authors focused on reviewing studies from recent years, while incorporating a combined retrospective by including some the most relevant membrane studies in the field conducted since the last decades. The review focused on an article search per topic using keywords. The authors collected, tabulated, and compared results from literature regarding state-of-the-art solid-liquid separation systems, feed characteristics for raw and digested manure, commercial membranes applied during separation of raw and digested manure, nutrient recovery methods using membranes and drawbacks of membrane application during treatment of farm effluents, among others. Additionally, the authors envisioned areas which need further investigations for implementing novel membranes and building sustainable processes during manure and digestates treatment. Data grouping, and comparisons were done when relevant, based on statistical analysis for average values and standard deviations, using the Microsoft Excel^®^ statistical analysis package.

## 3. Manure Treatment Processes

Manure treatment strategies have been developed to reduce water and air pollution from animal wastes. By doing so, nutrient recovery becomes possible and adds value to the entire manure management chain. The main manure treatment approaches consist of aerobic and anaerobic processes. In aerobic processes, bio-available compounds and nitrogenous compounds are oxidized decreasing the ammonia emissions [37]. Bacteria, protozoa and fungi are the main microorganisms degrading the organic matter and releasing CO_2_, H_2_O, and biomass as final products [38]. Approaches such as autothermal thermophilic aerobic digestion of liquid swine manure have also been reported [39], where the main benefits of this technology are the process simplicity, its robustness, the high reaction rate and the consequent small equipment, the conservation of N and the possibility of heat recovery. However, the applicability of this technology is limited. Anaerobic digestion (AD) is in fact recognized as the most sustainable and cost-effective technology for waste stabilization and production of valuable by-products like fertilizers and biogas [40,41].

### 3.1. Anaerobic Digestion

Anaerobic digestion has been successfully applied to different wastes [42,43] and manure sources, like poultry manure [44], pig manure [45], dairy manure [46], cow paunch manure [47], cattle manure [48], horse manure [49]. In general, AD follows four successive stages: hydrolysis, acidogenesis, acetogenesis, and methanogenesis with the overall process depending on the interaction of the microorganisms responsible for the different stages [50]. Digestate effluents are known as the material remaining after anaerobic digestion of organic wastes.

Compared to raw animal manure, the digestate has higher total ammoniacal-nitrogen (TAN) ratio, decreased organic matter content, decreased total and organic carbon, reduced biological oxygen demand, elevated pH, smaller carbon (C) to N ratio (C/N), and reduced viscosity [51]. From an agricultural point of view, parameters like pH, salinity, mineral N, especially in the form of ammonium, macro- and micronutrients, organic matter and concentration of heavy metals are important parameters when considering the application of digestates in agriculture [52].

Optimum C/N ratios for anaerobic digestion range between 20 and 30, with an optimal ratio of 25/1 for anaerobic bacterial growth in an AD system [53,54]. Improper C/N ratios could result in high TAN release and/or high accumulation of volatile fatty acids (VFA) in the digester, which would ultimately decrease the methanogen bacterial activity and cause failure of the AD process.

In the case of unbalanced C/N feedstocks, co-digestion of manure with different substrates increases the biogas production rate, improves the fertilizer value of the digestate and mitigates the greenhouse gas emissions [55,56].

The optimum biogas production during anaerobic digestion is achieved when the pH value of the ingestate is between 6 and 7. When the methane production level is stabilized, the pH range remains buffered between 7.2 and 8.3 [57]. Hartmann et al. [58], showed that during co-digestion of the organic fraction of municipal solid waste with cow manure, the pH rose to a value of 8 and the reactor showed stable performance with high biogas yield and low VFA levels.

Anaerobic digestion positively increases the agronomic value of the biomass treated by production of a digestate with a higher proportion of mineral N and less decomposable organic matter [59]. Masse et al. found that the ratio between TAN and the Total Kjeldahl Nitrogen (TKN) increased from 74% in raw manure to 85% in manure digestate. This was possibly due to the mineralization of organic nitrogen during anaerobic treatment [45]. Phosphorus (P) concentration in digestate represented 42% of that in raw manure [45,60]. However, the P availability results for AD are contradictory. Massé et al. [61] reported positive effects of AD in P availability, whereas Möller and Müller [51] maintained a more neutral position. Other authors found that co-digestates might be poorer in nutrients due to dilution [62], especially if the organic waste used for the co-digestion is low in nutrients or simply because of the addition of water into the anaerobic reactor to maintain a proper total solid content.

In relation to the reduction of particle size during AD, Møller et al. [63] found that the relative amount of swine manure particles smaller than 1.6 μm decreased from 27% to 10% during a seven-month storage period at 20 °C under anaerobic conditions. Masse et al. [45] also found that anaerobic treatment reduced swine manure solids concentration by 70% and significantly reduced the particle size. It was found that particles smaller than 10 μm represented 84% of dry matter (DM) for digested manure, whereas for raw swine manure these represented 64% of DM. Among particles smaller than 10 μm for anaerobic digestates, there were no particles larger than 2 μm detected. This suggests that, if a microfiltration step is intended for digested swine manure, the removal of particles larger than 10 μm may be sufficient as a pretreatment [64].

### 3.2. Solid-Liquid Separation (SLS)

When coupled with AD, manure SLS has been reported to add environmental benefits and increase the flexibility in manure management [65]. Manure separation processes aim at achieving a high separation efficiency in terms of concentration of suspended and colloidal particles in the solid phase, which is beneficial for further processing of the liquid fraction. Among the established treatment practices, solid-liquid separation of manure generates a solid fraction rich in P and a liquid fraction rich in N and K and, to lesser extent, in P [5].

Normally, centrifugation, gravity drainage and pressure filtration are used as manure pretreatment. Low-tech separation implies however a more difficult treatment of the liquid fraction due to the high amount of residual colloidal particles in the liquid phase. Most commercial solid-liquid separators can remove a considerable fraction of raw manure DM, but except for the decanter centrifuge, they are not efficient in terms of nutrient and heavy metal separation [66]. SLS can be performed before or after the AD. The separation before the AD process has the advantage of removing the material that hinders pumping and mixing. Nevertheless, part of the volatile DM is lost in the solid fraction decreasing the recoverable energy [67].

An overview of the main SLS techniques applied to manure separation with the DM from feed and obtained liquid fractions are summarized in Table 1. If the liquid fraction is further concentrated using membrane filtration, particles larger than 10 μm in anaerobic digestates would be removed during pretreatment of anaerobic digestates, as indicated previously by Masse et al. [45].

The removal of colloidal, suspended particles and soluble macromolecules from liquid fractions obtained after manure or digestate solid-liquid separation is possible using MF or UF [26,72]. If RO is applied, the recovery of concentrated soluble compounds such as ammonium, phosphates and potassium, apart from other ions in the concentrate is possible as well [27,28]. The advantage of using RO is the relatively large permeate flow achieved with a relatively small concentrate flow, while MF can be used as a pretreatment for RO [21,57]. The mineral concentrates obtained after membrane separation can be further post treated, as for example [5]:

- Precipitation of struvite (MgKNH_4_PO_4_) by increasing pH to over 9 through addition of Ca(OH)_2_, MgO or MgCl_2_ depending on which metal in the concentrate is limiting.
- Recovery of potassium by precipitating potassium struvite (KMgPO_4_), which occurs at high pH if the concentration of TAN is low and potassium, magnesium and PO_4_^3−^ are present in equimolar amounts.
- Evaporation of ammonia from the struvite precipitate by heating it and recovery of magnesium hydrogen phosphate for reuse
- Ammonia stripping by absorption in an inorganic acid or water
- Distillation and concentration of ammonia in a water phase
- Precipitation/crystallization of PO_4_^3−^ as calcium phosphate or calcium hydroxyl phosphate with the addition of Ca(OH)_2_

### 3.3. Combined Manure Treatment and Separation Strategies

**Figure 1:**
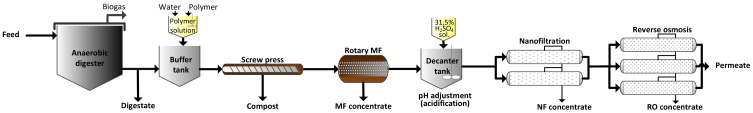
Post-treatment of digestate manure after solid liquid separation using screw press and different membrane technologies.

The synthesis of manure treatment processes implies a sequence of unit operations focusing on organic matter removal, nutrient recovery, solid separation, etc. As an example, Figure 1 shows the optimal solution proposed by Camilleri-Rumbau et al. [73] obtained by comparing different membrane technologies, physico-chemical operations and SLS techniques to recover nutrient-rich fractions from biogas digestate. The process was predicted to have a low energy consumption during solid-liquid separation and a low chemical consumption, thus proving the advantages of using membrane techniques to concentrate the nutrients present in digestate liquid fractions.

Table 2 gathers some essential literature about different manure treatment processes.

## 4. Raw Manure and Digestate Composition

Table 3 reports the composition and characteristics of pig manure as raw slurry and as separated liquid fraction. For the same manure, Table 4 summarizes the characteristics of the digestate before and after SLS. The raw manure composition gives fundamental information about the biogas potential achievable during AD, possibilities in land application or further treatment using membrane technologies for the liquid fractions. Similar information for other types of raw manures are provided in the Supplementary Materials Table S1.

### 4.1. Pig Slurry Composition

Pig slurry is a highly charged stream source rich in H_2_O, organic compounds (present in both suspended and colloidal particles); nitrogen compounds (mainly in ammonia/ammonium form), P, major cations and anions (K, Ca, Mg, Na, Cl and sulphate); heavy metals (mainly Cu, Zn and Cd) and organic pollutants such as weeds, pathogens, medicine residues and residues of pesticides, herbicides and fungicides [60].

According to Table 3, the pH of raw pig slurry varies between 6.7 and 8.35 depending on the storage period, application of buffers and temperature [77]. As expected, total solids (TS) concentration is reduced mainly after manure solid-liquid separation treatment. Decanter centrifugation presents a higher TS separation efficiency than the one observed for screw press (approximately 67% and 38%, respectively) [5,26,45,60,73]. TS can also be reduced by about 70% using straw filtration [60]. In terms of nutrient recovery, approximately 78% of the P contained in the raw slurry can be recovered when applying straw filtration and around 83% by microfiltration. Masse et al. [45] suggested that in digestates and raw manure, approximately 20% of total P is in soluble form, whereas another 50% is associated with particles between 0.45 and 10 μm. They reported that only 30% P is linked to particles larger than 10 μm. About 80% P in swine manure is linked to suspended solids mostly attached to particles within 0.45–250 μm diameter.

In relation with DM content, Westerman et al. [70] showed that there was little difference between the concentration values of flushed wastes from finishing pigs and the screened liquid. Concretely, they showed that after separating manure by screw press, fine screening and dewatering the resulting fraction, the separated solids still contained almost 40% of DM and a high nutrient value (about 18 mg N, 10.4 mg P and 4.6 mg K per gram of DM). While total suspended solids (TSS) and TS decreased by about 20% and 13.5%, respectively after screening, while nutrient concentrations in the screened liquid remained almost unchanged.

### 4.2. Anaerobic Digestate Composition

Table 4 presents the composition and characteristics of anaerobically digested manure. It can be observed that pH values remain between 8.01–8.30 for all digestate sources. However, a lower pH value of 7.2 was detected by Masse et al. [45] during psychrophilic dry anaerobic digestion of dairy cow manure. TS were reduced by about 50% after applying decanter centrifuging to digestates [66]. Ammonia remained between 3.3–5.9 g·kg^−1^ in digestates, with minimal effect from separation [26,67]. K was mainly found as dissolved K^+^ remains also practically unchanged after solid-liquid separation with values between 2.0 and 3.0 g·kg^−1^. P in separated digestates, being mainly bound in particulate matter, decreased from 0.78–1.67 g·kg^−1^ to 0.21–0.67 g·kg^−1^ [26,63] when using decanter centrifuge or screw press. Camilleri-Rumbau et al. [26] further found that microfiltration could recover about 80% of the P present in digestate liquid fractions.

As can be observed from the data presented in Table 3 and Table 4, mechanical separation greatly influences manure and digestate composition. As reported by Masse et al. [45], a decanter centrifuge is one of the most efficient solid-liquid separators in terms of nutrient and heavy metal separation. In general, a decanter centrifuge removed all particles > 2 μm [23] while a screw press mainly retained particles > 1 mm [63]. Additionally, mainly the smaller particles are degraded during the anaerobic digestion leading to a relative increase in the proportion of larger particles (>1.4 mm) [78]. A particular separator may be found superior to another based on testing that alters screen size, flow rate, or influent manure DM concentration. However, factors such as power requirements and cost have to be taken into account to evaluate the techno-economic performance of these separators [79].

### 4.3. Heavy Metal Composition

In the livestock production industry, it is a common practice to use Zn and Cu supplements in animal feed due to their growth-stimulating and antimicrobial effects [80,81,82]. Lowering the dietary supply of these elements to the livestock would be the most effective way to control heavy metal contents in digested manure slurries [82]. However, the removal of heavy metals from wastewater sources is a major concern mainly due to abrupt interference with the environment, bioaccumulation and related health risks [83]. After thermal treatment of manure, Li et al. [84] found that 75–90% of heavy metals such as Cr, Ni, and Mn are mainly found in the solid-phase, while heavy metals such as Cd, As, Hg, and Pb are found in the aqueous phase and gas phase, accounting for less than 5% of their total concentrations.

Despite the fact that animal manures are likely to contain high levels of heavy metals that pose risks to the environment and to human health, the addition of certain metals to the feed material has also been found to increase biogas production [85]. It has been demonstrated that efficient removal of propionate at high levels of VFA requires supplementation of Ca, Fe, Ni, and Co in a thermophilic non-mixed reactor [86]. Masse et al. [45] reported that approximately 80% of Zn and more than 95% of Cu in anaerobic digestate swine manure were associated with particles between 0.45 and 10 μm. Jin and Chang [82] found that total concentrations of Zn, Cu and As in digested pig slurries were <10, <5 and 0.02–0.1 mg·L^−1^, respectively. Low concentration of Cu and Zn are also present in screened liquid slurries (8.5 and 11.2 μg·g^−1^ dry basis, respectively) [70]. Leclerc and Laurent [87] presented a global compilation of national release inventories for heavy metals considering 215 countries during a 15-year period. They found that mercury, zinc and copper are mostly responsible for the toxic impacts on human health and freshwater ecosystems resulting from manure application to land. Further information about the composition of manure and digestates in terms of heavy metals is shown in Table 5.

Although it is well-known that heavy metals are present in animal manure and can be concentrated during manure treatment, there is limited literature on membrane technology and the effect of heavy metals concentration [88].

**Table 3:** Composition of raw pig slurry and separated raw pig slurry.

Raw Pig Slurry									
pH	TVS [g·kg^−1^]	COD [g L^−1^]	TS [g·kg^−1^]	DM [g·L^−1^]	TKN [g·kg^−1^]	TAN [g·kg^−1^]	P [g·kg^−1^]	K [g·kg^−1^]	Reference
NA	-	NA	NA	67 ± 26	7.5 ± 2.5	4.5 ± 2.1	2.1 ± 0.8	3.3 ± 1.1	[5]
NA	-	NA	NA	18.4 ± 0.7	NA	2.06 ± 0.19	0.65 ± 0.13	2.32 ± 0.33	[21]
7.7 ± 0.1	-	45.01 ± 3.20	NA	45.5 ± 3.1	6.7 ± 0.5	5.5 ± 0.4	1.6 ± 0.2	2.6 ± 0.2	[28]
7.32 ± 0.16	74.72 ± 12.67	NA	95.84 ± 15.22	NA	10.49 ± 1.56	7.72 ± 1.11	2.49 ± 0.30	4.83 ± 0.80	[45]
7	-	-	NA	73	6.3	NA	1.62	5.98	[57]
8.5 ± 0.2	42 ± 11	NA	61 ± 11	-	7.0 ± 0.5	3.5 ± 0.5	1.8 ± 0.2	5.5 ± 0.5	[60]
7.4 ± 0.2	34 ± 18	NA	51 ± 22	-	5.1 ± 1.2	3.5 ± 0.6	1.2 ± 0.6	4.7 ± 0.7	[60]
7.5	-	45.96	NA	53.2	4.2	3.6	1.26	3.2	[63]
7.09 ± 0.10	36 ± 2	26.00 ± 3.10	48.0 ± 1.8	NA	5.6 ± 0.1	4.8 ± 0.7	1.6 ± 0.05	NA	[66]
6.72	-	NA	NA	34.5	9.35	3.66	0.74	3.62	[77]
8.35 ± 0.23	8.43 ± 6.54	NA	14.87 ± 9.56	NA	NA	3.033 ± 1.12	0.19 ± 0.16	NA	[89]
NA	-	-	26.9	NA	2.12	1.46	1.46	1.36	[90]
Separated raw pig slurry									
8.3 ± 0.2	-	40.32 ± 1.93	-	15.6 ± 0.5	NA	5.1 ± 0.5	0.24 ± 0.02	2.5 ± 0.3	[28] ^1^
8.4± 0.2	8.0 ± 2.6	NA	19 ± 0.0	-	4.7 ± 0.8	2.7 ± 0.4	0.4 ± 0.3	4.9 ± 0.5	[60] ^2^
7.8 ± 0.2	8.4 ± 1.8	NA	18 ± 3	-	3.4 ± 0.4	3.1 ± 0.3	<0.2	4.6 ± 0.5	[60] ^3^
8.01 ± 0.12	-	9.433 ± 0.472	14.49 ± 0.72	-	1.71 ± 0.068	1.41 ± 0.071	0.20 ± 0.01	NA	[72] ^4^
6.76 ± 0.03	-	-	NA	6.10 ± 0.49	NA	1.66 ± 0.07	0.06 ± 0.0	1.09 ± 0.05	[91,92] ^5^

Values are expressed as average ± standard deviation. NA: not available. All values in g·kg^−1^ considering that slurries and digestates have a density of about 1kg·L^−1^ [5]. ^1^: data obtained for vacuum filtration; ^2^: data obtained for straw filter made of three-layered plastic foil, total tickness 0.2 mm; ^3^: data obtained for the sequence screw press, decanter centrifuge and MF; ^4^: data obtained for liquid filtered by a 0.5 mm screen. ^5^: data obtained for belt separator, decanted and passed through a 800 μm bag.

**Table 4:** Composition of anaerobic digestates.

Digestate before Separation								
pH	TVS [g·kg^−1^]	TS [g·kg^−1^]	DM [g·L^−1^]	TKN [g·kg^−1^]	TAN [g·kg^−1^]	P [g·kg^−1^]	K [g·kg^−1^]	Reference
8.24 ± 0.23	16.20 ± 3.74	28.29 ± 5.87	NA	6.9 ± 1.08	5.89 ± 0.73	1.04 ± 0.34	2.99 ± 0.31	[45] ^1^
8.3	-	NA	56.2	NA	4.2	0.89	2.54	[63] ^2^
8.1	-	NA	65.3	NA	5.0	1.67	2.31	[63] ^3^
8.1	-	NA	35.5	NA	3.8	1.11	2.71	[63] ^4^
8.01 ± 0.11	16.2 ± 1.01	48.1 ± 1.9	NA	5.3 ± 0.08	3.7 ± 0.4	1.5 ± 0.05	NA	[66] ^5^
Effluent after digestate separation								
8.2±0.1	-	NA	27	3.4	3.15	0.46	2.03	[26] ^6^
NA	-	NA	52.3	3.80	NA	1.17	NA	[63] ^7^
NA	-	NA	19.6–24.4	3.1–5.2	NA	0.21–0.51		[63] ^8^
8.09 ± 0.10	-	21.0 ± 0.9	NA	4.3 ± 0.08	3.5 ± 0.4	0.6 ± 0.05	NA	[66] ^2^

Values are expressed as average ± standard deviation. NA: not available. ^1^: manure from growing finishing swine operation treated in anaerobic sequencing batch reactors at 25 °C; ^2^: mix of 75% pig manure and 25% other waste fish-processing waste continuous AD in a thermophilic (53 °C) apparatus; ^3^: mix of 75% pig manure and 25% other waste fish-processing waste continuous AD in a mesophilic (35 °C) apparatus; ^4^: mix of 98% pig manure and 2% fatty waste continuous AD in a mesophilic (38 °C) apparatus; ^5^: mix of 90% pig manure and 10% fish-processing waste continuous AD in a thermophilic (55 °C) plant; ^6^: liquid fraction obtained through decanter centrifugation; ^7^: obtained with screw press of the digestate of 2; ^8^: obtained with decanter centrifuge of the digestate of 3 and 4 respectively.

**Table 5:** Typical heavy metal concentrations in raw and digestate manure.

Source	Zn [mg/L]	Cu [mg/L]	Fe [mg/L]	As [mg/L]	Solid-Liquid Separation Technique	Reference
Raw pig manure slurry	12.75 ± 1.65	7.54 ± 2.96	-	0.13 ± 0.10	None	[82]
Digested pig slurry	20.66 ± 6.99	16.30 ± 4.1	-	0.26 ± 0.14	None	[82]
Solid fraction of digested pig slurry	477 ± 40.4	204 ± 30	-	2.19 ± 0.88	Sedimentation in anaerobic digester	[82]
Digested swine manure (for particles < 10 μm)	45.90	19.68	71.70	-	None	[45]
Digested swine manure (for particles < 0.45 μm)	2.17	1.07	4.20	-	None	[45]
Manure co-digestate ^1^	16.4	6.4	1099	-		[93]
Digested swine manure	64 ± 2.00	9.05 ± 0.1	-	-		[94]
Pig biogas slurry ^2^	9.88 ± 2.1	2.74 ± 0.45	-	-		[95]
Co-digestate pig slurry	24.6 ± 4.2	4.8 ± 0.7	-	-	Decanter centrifuge	[68]
Co-digestate liquid fraction of pig slurry	27	5	46	-	Decanter centrifuge	[19]
Co-digestate liquid fraction of cow manure	5	1.4	208	-	Decanter centrifuge	[20]

Values are expressed as average ± standard deviation. ^1^: digestate obtained from animal manure, energy maize and food industry residues. ^2^: pig manure anaerobically digested. The digestate is stored for 45 days and separated by natural sedimentation in biogas slurry and biogas residue.

## 5. Membrane Technologies for Post-Treatment of Manure and Digestate Liquid Fractions

After solid-liquid separation of manure or digestate, the obtained liquid fraction can be further concentrated using membranes. One of the main limitations of using membranes is membrane fouling, which represents one of the major problems during processing since it limits the membrane continuous operation. Several parameters can affect membrane fouling severity. Flow conditions, membrane pore size and/or selectivity, ion rejection capacity, membrane material, physico-chemical properties, porosity and morphology of the surface [96], as well as the characteristics of the effluent being treated are the main parameters contributing to fouling. Understanding the fouling mechanisms involved during concentration of livestock manure liquid fractions is one of the main challenges during the application of this technology.

### 5.1. Membrane Classification and Material Properties

Previous research studies on application of membranes during manure and digestate liquid fractions treatment have mainly used MF, UF, RO, membrane contactors (MC) and ED. Introduction of FO in this field is a relatively new method.

The selection of membrane material plays a very important role in separation performance during farm effluent processing. Membrane performance is mainly linked to physical and chemical interactions between the membrane surface and foulants during processing of water and wastewater sources. Material characteristics such as material type, porosity, hydrophobicity-hydrophilicity, surface charge, membrane polarity, permeability, selectivity, etc. are important factors affecting membrane performance. Table 6 presents some of the most used commercial polymeric membranes for the treatment of farm effluents. Polymeric membranes are typically made of polysulphone (PS), polyethersulphone (PES), polyvinylidene fluoride (PVDF), polypropylene (PP), polytetrafluoroethylene (PTFE), cellulose acetate (CA), cellulose triacetate (CTA), polyamide thin film composite (PA), while inorganic membranes typically are based on aluminum oxide or titanium oxide.

Membrane hydrophobicity or hydrophilicity influences membrane fouling. Hydrophobic membranes are widely reported to be more susceptible to adsorptive fouling by organic particles than hydrophilic ones [97]. Zhang et al. [98] found that the adsorptive fouling degrees were in increasing order PAN < PVDF < PES. Camilleri-Rumbau et al. [26] reported that in the initial stage of the digestate liquid fraction concentration process, PS membranes had a higher fouling tendency than PVDF membranes. However, after cake layer formation, the influence of the membrane material became less relevant and the cake layer controlled the filtration mechanism. Studies from Boerlage et al. [97] also demonstrated that PS membranes had a higher tendency to foul compared to polyacrilonitrile (PAN) membranes, as expected due to the higher hydrophobicity of PS compared to PAN. Furthermore, in their study, a homogeneously permeable surface enhanced particle deposition and formation of a regular cake layer, which was easier to remove with cleaning regardless of the low surface porosity detected [97].

López-Fernández et al. [72] further found that PES had a higher tendency to foul than PVDF. The permeability of PES membranes decreased drastically by 93%, while the permeability on PVDF membranes decreased by around 25%. This was possibly due to the higher affinity of the extracellular polymeric substances to the PES membrane [98]. The same study reported that the selectivity of the UF membranes filtering swine manure liquid fractions was directly related to the membrane rejection and the selected operating conditions. It was found that the increased permeate flux when using PVDF membranes improved the filtration selectivity, with higher COD rejection values for PVDF membranes compared to PES membranes (70% and 55–70%, respectively). Boerlage et al. [97] also reported the importance of the membrane material on the fouling mechanism. It was suggested that fouling observed on PAN membranes was dominated by physical deposition while fouling observed on PS was more likely chemically adsorbed, due to the more hydrophobic nature of PS. During MF of digested swine manure, Camilleri-Rumbau et al. [26] observed that permeate flux decline initially occurs due to a fast fouling formation followed by a filtration period with a slower relatively constant flux decline. This study further showed that foulants were adsorbed stronger to the PS membrane surface than to PVDF membrane surface. Similar conclusions were obtained in other studies using the same type of membrane materials [90,98].

Apart from the influence of membrane material during the filtration process, high porosity of membrane surfaces can also enhance fouling [99], due to the increased flux across the membrane which will drag more foulants towards the membrane surface and hence could provoke pore blocking. The effect of membrane charge and polarity, measured as electrical conductivity and ion exchange capacity, during separation of manure compounds such as NH_3_-N is of special relevance when using ED. Mondor et al. [28,100] used AMX anion-exchange and CMB cation-exchange membranes to isolate and concentrate total NH_3_-N from swine manure, achieving five-fold concentrations compared to feed manure. However, the main drawbacks of this technique were fouling of the AMX membranes mainly from calcium carbonate and silica, and consequently a loss in stack average current density. Fouling on CMB would however be minimal due mainly to electrostatic repulsion with Ca^2+^ ions and the negatively charged silica colloidal particles.

### 5.2. Recovery of Total Ammonia Nitrogen (TAN) 

Approximately 70% of the N in raw pig slurries is dissolved and present as ammonium while the rest is bound to particles such as organic macromolecules, proteins and inorganic precipitates. At a pH higher than 8, TAN is mostly present in manure as uncharged ammonia, due to the existing equilibrium between ammonium (NH_4_^+^) and ammonia (NH_3_). Contrarily, at low pH, the TAN is mostly present as positively charged ammonium (NH_4_^+^) [20]. Based on the ammonia-ammonium equilibrium chemistry, there here have been different approaches on ammonia recovery from animal waste in literature.

In digestates, for instance, ammonia recovery can be done by adding sulfuric acid to the liquid fraction obtained after mechanical separation of solids, as a treatment step after phosphorus recovery as calcium phosphate or struvite precipitate. Using concentrated sulfuric acid would volatilize CO_2_ and capture ammonium, which would be stable at this pH, as an ammonium sulphate solution [57]. Recovery of nitrogen by ammonia stripping is also possible and it requires however a partial increase of pH in the stripping phase [57]. By neutralizing the stripped liquid fraction, RO can be applied for obtaining a concentrate with low concentration in ammonia and phosphates and a high concentration in K, and possibly some small amount of precipitate [6].

During ammonia recovery using RO membranes, the rejection of ammonium is higher than its uncharged form (ammonia), where rejection depends strongly on the pH [27,100]. RO membranes have proven to be able to recover more than 99% of the TAN present in raw slurry at pH < 6.5 [27]. At a similar pH level, a TAN rejection higher than 95.5% could also be achieved using aquaporin-based forward osmosis membranes on digested manure [20]. However, Li et al. [88] found FO ineffective in retaining N species. This result could be attributed to volatilization from the feed solution or N attachment on the membrane surface.

MD has also been studied as an ammonia recovery technique from raw swine manure. One of the main drawbacks of this technique is membrane wetting due to fouling formation, which provokes a loss in hydrophobicity and hinders the ammonia stripping capabilities of the membrane [101]. However, pretreatment methods such as MF and UF showed to be effective in enhancing the ammonia mass transfer coefficient, concretely by two in the case of PTFE membranes and by four in the case of PP membranes [101].

Ammonia recovery using ED has previously been applied in swine manure [101,102]. Mondor et al. [28] further considered a combination of ED and RO in treating liquid swine manure pretreated by vacuum filtration. The system showed promising results, but the possibility of ammonia volatilization represented a challenge to be taken into account. The authors reported that after 10 ED batches, approximately 17% of NH_3_ was volatilized. In order to minimize nitrogen losses during RO processes, pumping, storing, increasing temperature, etc. it is suggested to use a closed system. However, ammonia emissions are more likely to occur during subsequent storing of the concentrate rather than before the concentration process [6].

Garcia-Gonzalez and Vanotti [103] have grouped the technologies for N recovery in five categories:

(1) RO using high pressure and hydrophilic membranes,
(2) air-stripping using stripping towers and acid absorption,
(3) zeolite adsorption through ion exchange,
(4) co-precipitation with phosphate and magnesium to form struvites,
(5) gas-permeable membranes at low pressure.

In the first category, Zhou et al. [104] developed a pre-treatment system composed by a sequence of sand filter, steel plate and frame filter, ceramic UF membrane before a RO module. They consider biogas slurry feedstock odtained from chicken manure. Single and double disk tube RO (DTRO) modules were used in combination with seawater RO (SWRO). The authors optimized the slurry pH, operating temperature and pressure to maximize the ammonia recovery. The dual DTRO-SWRO at the optimized conditions (pH 6.1, 5 MPa, 35 °C) reached 99.1% TAN rejection.

In the second category more traditional technologies based on stripping performed in towers are included. Ammonia is stripped from pre-treated manure using air, steam or biogas and then absorbed in an acid solution to produce added-value fertilizers [105,106,107]. The modelling of this systems represents a challenge as highlighted for an analogous system by Madeddu et al. [108,109].

The third category zeolites can be added to both manure or sewage sludge-based digestate solid to provide active sites for molecular adsorption or exchangable cations for ammonium ions. Both mechanisms can increase the N retention [110]. The formation of struvites, included as forth category is discussed in the Section 4.3 together with the phosphorus recovery. In the last category, NH_3_ pass through a microporous hydrophobic membrane and is concentrated in a stripping solution on the other side of the membrane. Garcia-Gonzalez and Vanotti [103] applied this technology to liquid swine manure using submerged tubular expanded polytetrafluoroethylene membranes. A H_2_SO_4_ solution was used to circulate acid through the membrane. The authors observed that adjusting the manure pH to 9 the TAN removal efficiency reached 88–94%. The same system was used to study the effect of other parameters like the aeration on the removal efficiency [111,112].

### 5.3. Recovery of Phosphorus

The liquid fraction obtained after mechanical separation of manure is rich in soluble components as well as colloidal and suspended particles. Colloidal and suspended particles contain insoluble organic and inorganic compounds such as phosphate or other P components, Ca and Mg precipitates.

Schoumans et al. [57] reviewed and assessed treatment strategies with regards to P recovery from manure and sewage sludge. Some of these strategies involve recovery of P_2_O_5_ in ash after incineration, recovery of phosphate as calcium phosphate or struvite and P-biochar after pyrolysis from which resulting pyrolytic oils (tar) and gases could be used for producing energy during pyrolytic process. Phosphates could be obtained from the sludge liquid fraction by precipitation with aluminum sulphate, although this method seems uneconomical because the entire liquid fraction has to be treated. In this regard, Christensen et al. [113] observed that about 70–90% of the total phosphate in raw pig slurries was found in the particulate fraction while the remainder occurred as dissolved phosphate.

A possible pathway for P recovery is its precipitation in the form of struvite by co-precipitation of ammonium, potassium and phosphate [114]. However, the formation of struvite requires a sufficient amount of phosphate, with the disadvantage that the solubility of struvite in water is relatively high [5]. Struvite precipitation takes place during anaerobic reaction at a pH of 8.3 approximately. The formation of struvite can be enhanced by addition of dissolved magnesium. After centrifugation of the digestate, the solid fraction, with approximately 80% of the total P, can be sent to composting while the liquid fraction could undertake several pathways in order to recover ammonia/ammonium as a product (see Section 4.2. Recovery of TAN) [57]. Karakashev et al. [66] also presented a system for P recovery via struvite precipitation coupled with anaerobic digestion, achieving a phosphate removal of 96%, 7% ammonium removal, a slight decrease in COD, while practically no change in TS and TSS was achieved. Maurer et al. [115] also reported that recovery of P by struvite formation was highly effective (P recovery rate of 90–100%); by applying volume reduction processes such as evaporation, freeze-thawing and electrodialysis prior to the P recovery step.

Phosphorus recovery using membranes has also been well documented. Camilleri-Rumbau et al. [19,26] used MF and UF membranes for treatment of digested manures. They found that more than 80% of the total phosphorus could be rejected using PS and PES membranes. Some authors have used pretreatment strategies of the liquid fraction to achieve precipitation of P and its removal together with colloidal and suspended particles. Christensen et al. [113] suggested that pH control could be used to regulate the concentration of dissolved P in manure and as precipitated struvite. The addition of CaO, MgO or Ca(OH)_2_ would care an increase in pH and promote precipitation of phosphate as calcium phosphates or calcium carbonates [5], while ammonia could be removed by stripping of the subsequent UF permeate.

Pramanik et al. [116] used a flat sheet FO membrane to pre-concentrate anaerobically treated dairy manure. The authors tested NaCl, MgCl_2_ and EDTA-2Na as possible draw solutions obtaining in all cases more than 98% PO_4_^3−^ rejection. However, supersaturation of different chemical species close to the membrane surface can cause membrane fouling. For this reason, Shi et al. [24] investigated using electrodialysis reversal (EDR) where the polarities of the electrodes are frequently inverted inducing a self-cleaning mechanism. The system was tested with a pre-treated and acidified pig manure digestate using cation and anionic membrane sheets. The authors measured a removal of phosphate up to 84%.

### 5.4. Rejection of Heavy Metals

Masse et al. [45] reported that in anaerobic digestates from swine manure 80% of Zn, Ca, and Fe and over 95% of Cu approximately are associated with particles between 0.45 and 10 μm. Several treatment strategies for the removal of heavy metals have been reported in literature. The addition of lime is used to promote precipitation of heavy metals, such as Cu and Zn hydroxides that can be efficiently removed from flushed raw swine manure waste [70]. During membrane separation, micellar-enhanced UF (MEUF) has also shown be a viable technique for separating phosphates from heavy metals, such as Cd and Cu, since P was not retained by the micelles and passed through the UF membrane [117]. Rejection coefficients up to 98% were achieved for both metals when no P was present, using sodium dodecyl sulfate (SDS) as a surfactant. Camilleri-Rumbau et al. [19] found that 96.9% Cu, Zn, Fe, Ca, Mg and Al could be rejected during the UF of centrifuged digestate manure, regardless of the use of flocculation-coagulation during the solid-liquid separation by centrifugation. FO aquaporin and conventional polyamide TFC FO membranes were tested by Li et al. [88] for digestate centrate liquid fraction obtained by natural settling. For both membranes the authors observed a rejection of heavy metals higher than 80%. Recently, Fernandes et al. [118] proposed a membrane sequence of MF + UF/NF for processing the digestate from kitchen and food waste. They observed that Al was reduced by 81% after MF and the NF was ineffective in Al rejection. When UF was used after MF, the Al was reduced by 87% compared to the pre-treated digestate. Zn was removed by 97% after MF without further improvement by adding the UF or NF step. Fe was reduced by 63% by the system MF-NF. The authors highlighted NF was the only process that produced a colorless permeate.

**Table 6:** Classification of the main commercial membranes applied for separation of manure and digestate liquid fractions treatment.

Technique	Pore Size [nm]	Permeability [L·m^−2^·h^−1^·bar^−1^]	Applied Pressure [bar]	Membrane Material	Module Configuration	Feed	Rejection	References
MF	100–10,000	>1000	0.1–2.0	PS, PVDF, Al_2_O_3_, monolith ceramic membrane	Flat sheet Submerged capillary	Mainly digestate Raw slurry	Particles	[21,26,60,66,119]
UF	2–100	10–1000	0.1–5	PS, PES, PVDF, coated cellulose, inorganic-silicon carbide, cellulose acetate	Tubular Submerged hollow fiber	Mainly digestate	Suspended solids, particulate phosphorus, nitrogen, COD, 99% coliforms; soluble COD, phosphates or nitrogen require a biological step to be removed; other macromolecules and multivalent ions in a lesser extent Note: Concentration factors between 1.7 and 9 *v*/*v* depending on the pretreatment used before the membrane step	[19,27,71,72,119,120,121]
NF	0.5–2	1.5–30	3–20				Particles, macromolecules, multivalent ions and small organic compounds in a lesser extent	[115]
RO	99% salt rejection	0.05–1.5	5–120	Polyamide thin film-composite Cellulose acetate	Flat sheet Spiral wound	Mainly raw slurry	Particles, macromolecules, monovalent and multivalent ions and small organic compounds	[21,27,91,92,122,123,124,125,126,127,128]
FO	99% salt rejection	>7 (in FO mode)	0 (just residual pressure from flow velocity)	Polyamide thin film composite (TFC)	Flat sheet plate and frame Spiral wound Hollow fiber Tubular	Digestate liquid fraction Digestate from municipal wastewater	Particles, macromolecules, monovalent and multivalent ions and small organic compounds	[20,88,116,129]
MC	100–10,000	NA	NA	PP PTFE PVDF	Flat sheet Tubular Hollow fiber	Raw pig slurry and digestate (from municipal waste)	Monovalent and multivalent ions	[29,101,102]
ED	Typically 200 Da apparent pore size	NA	NA	Anionic/ cationic membranes	Electrodialysis cell	Mainly raw slurry (or urine source wastewater)	Monovalent and multivalent ions	[28,100,115,130]

## 6. Membrane Fouling and Factors Influencing Membrane Performance

Manure is a complex wastewater source with a high membrane fouling propensity. Swine manure, for instance, is a highly complex mixture of inorganic colloids and suspended organics, volatile fatty acids, proteins, bacteria, suspended solids and inorganic elements including Ca, NH_4_^+^, Na, S, K, P and Mg [33,131,132]. Ruan et al. [133] reported that the main composition of the inorganic fouling found on RO membranes processing digestate slurry was mainly Ca (as CaCO_3_ and CaSO_4_), Si (as SiO_2_ colloid), O, C, Cl, Na, S, and P as well as organic foulants based on complex components, including hydrocarbons, aliphatic acids and their analogues.

Effluents with high ionic strength have been previously correlated to increased fouling [134,135] and cause more rapid flux decline [136], being membrane scaling and fouling the main factors affecting membrane performance [137].

The importance of membrane fouling during manure processing with membranes has been addressed in the last years. Reverse osmosis membranes has been studied previously for membranes treating swine wastewater [92,122,124,131] and other wastewater sources [23]. Fouling has also been studied in MF and UF systems [19,26,72,120,138,139] treating anaerobically digestated slurries; in membrane contactors for ammonia stripping from pig slurry [101,102] and in ED membranes during concentration and recovery of ammonia from swine wastewater [100]. Studies on fouling of forward osmosis membranes for processing manure wastewater sources are also limited [20].

Several factors have been attributed in the literature to be the main cause for membrane fouling and membrane performance loss. This section gives an overview of literature describing fouling mechanisms, operating conditions, cleaning and ageing of membranes processing mainly raw and digestate manure. An overview of membrane and fouling characterization techniques is available in the Section S1 of the Supplementary Materials.

### 6.1. Membrane Fouling Mechanisms

Membrane fouling mechanisms are complex and thus difficult to define. Several authors have put efforts in explaining the related mechanisms for specific applications. For instance, Lim and Bai [140] suggested that during MF of activated sludge the fouling mechanism could initially be based on “membrane-limited fouling”, followed by “pore blocking”, and eventually “cake formation”. Other authors explain fouling mechanisms based on an initial concentration polarization, followed by gel layer formation and finally cake layer formation [141].

The use of models to explain the complex mechanisms taking place during membrane fouling is thus of importance to understand the filtration data. The resistance in series model as shown in Equation (1) is the calculation method most often used for evaluation of membrane permeate flux and fouling resistance:

(1) $$J_{p}=\frac{\Delta P-\Delta \pi }{\mu \left(T\right)\cdot R_{t}}$$

where *J_p_* (m·s^−1^ or L·m^−2^·h^−1^) is the permeate flux through the membrane, Δ*P* (Pa or bar) is the applied pressure, Δ*π* (Pa or bar) is the osmotic pressure, *μ*(*T*) is the viscosity of the fluid at a given temperature and *R_t_* (m^−1^) is the total resistance of the fouling layer-membrane system which acts as a barrier to the permeate flux.

The total resistance *R_t_* can be divided into a pure membrane resistance and reversible and irreversible fouling contributions. The classification of membrane total resistance can be defined as shown in Equation (2). However, there is not a universal agreement on this classification and authors use different contributions in their definition of *R_t_* and definitions for reversible and irreversible fouling [125,139,142,143]:

(2) $$R_{t}=R_{m}+R_{s}+R_{f}$$

Camilleri-Rumbau et al. [91,92] classified *R_t_* from RO filtration data from raw swine wastewater, as the sum of *R_m_*, *R_s_* and *R_f_* (Equation (2)). *R_m_* was defined as the intrinsic membrane resistance to water flux, which may increase as the membrane is put into use, due to compaction and irreversible fouling. The substrate resistance (*R_s_*) was attributed to specific membrane-solute interactions which might occur even in the absence of flow. Fouling resistance (*R_f_*) was the result of a variety of phenomena such as concentration polarization, gel layer formation and cake layer formation on the membrane surface. Several authors related the rapid increase in *R_f_* during swine wastewater processing, to the transition between the pressure-dependent flux region and the pressure-independent flux region (i.e., gel-polarized region) [91,92].

Fouling mechanisms for membrane processes using MF/UF/NF/FO/ED/MD are more difficult to be identified. In this regard, authors normally refer to studies on municipal wastewater applications to explain the observations during the filtration of manure effluents.

### 6.2. Permeate Flux Decay and Pressure Control 

Following Darcy’s law, pure water fluxes in pressurized systems are proportional to the operating pressure applied, regardless of the variation on cross-flow velocity. However, during membrane processing of highly charged wastewater streams, deviations from linearity occur due to the presence of foulants. For instance, the water flux for clean UF membranes can be between 600–1000 L·m^−2^h^−1^, while during manure filtration the flux is reduced to 20–40 L·m^−2^h^−1^. This reduction might be explained by gel polarization and cake formation phenomena [115]. However, turbulence on the membrane surface, although generally helps to remove/lift foulants on the membrane surface, could also promote the inclusion of small particles in the cake layer. This could result in a less porous layer and higher specific resistance resulting in a lower permeate flux [136]. In general, compact smooth biofilms are formed at high shear force, while thick, fluffy biofilms are produced at low shear force [134,144]. Further increasing the applied pressure results in a denser and more compact cake layer which increases the specific cake resistance.

During MF of digestate liquid fractions, Camilleri-Rumbau et al. [26] observed that an increase in the feed cross-flow velocity increased the permeate flux on PS and PVDF membranes. This was attributed to concentration polarization and a reversible fouling layer that could be removed when increasing turbulence at the membrane surface. However, combining a higher pressure with a low cross-flow velocity resulted in the lowest permeate fluxes probably due to a significant increase in the fouling layer resistance caused by increased compression of the fouling layer [115]. Similar observations were obtained during UF of pretreated raw pig slurry by Fugère et al. [71]. They found that the optimal operating parameters during UF of pretreated raw pig slurry remained similar even though manure compositions and pretreatments were different. The authors explained this by the major contribution of fouling by gel-cake layer formation, as gel-cake layer formation is influenced more by the hydrodynamic conditions at the membrane surface than by the solids concentration in each manure type. Waeger et al. [138] also suggested the dominance of cake layer formation over permeate flux decay, during processing of anaerobic digestate liquid fraction. Therefore, increases in cross-flow velocities instead of the applied pressure might sometimes benefit the permeate flux rate [120,138], athough higher velocities also imply a higher energy consumption.

### 6.3. Influence of Manure Composition and Particle Size

As discussed in Section 3, raw and digestate liquid fractions are highly charged wastewater streams rich in particles, proteins, colloids, nitrogen, phosphorus, potassium and metals, among other elements. In particular, the influence of metals during membrane processing cannot be neglected, as non-bound metals can cause severe membrane flux decay. Divalent cations, especially Ca, can form strong complexes with organic matter and polysaccharides, such as extracellular polymeric substances (EPS), resulting in the formation of a compact cake layer highly resistant to hydrodynamic forces [131,145]. Approximately 95% Fe and 80–90% Ca and Mg were found in the particulate fraction in raw pig manure [113], while approximately 80% of Zn, Ca, and Fe and over 95% of Cu were associated with particles between 0.45 and 10 μm in anaerobic pig digestate. As both Ca and Mg may form complexes with dissolved organic matter, the amount of ionic Ca and Mg may be 10–20% of the total concentration in the dissolved fraction [113,146].

In a study comparing different wastewater streams including cattle and pig slurry, Reimann et al. [137] found that the permeability of UF and RO membranes is determined by the concentration of organic matter in the wastewater and is independent of the type of wastewater. They reported a permeability loss over 83% when the COD-concentration increased from 0.63 g·L^−1^ to 42.8 g·L^−1^. However, Fugère et al. [71] found that COD decreased significantly in raw manure with longer hydraulic retention time since allowed manure biodegradation which resulted in smaller particles. High organic matter concentrations together with the formation of bigger flocks in fresh filtered manure, make manure more difficult to treat, thus leading to a faster membrane flux decrease [71].

Particle size and particle concentration play an important role during membrane operations processing wastewater effluents. Studies show that the higher the feed concentration, the more the permeate flux decline is expected. Additionally, the cake resistance increases with the feed concentration although the specific cake resistance is not influenced and only the cake layer thickness is increased. Masse et al. [45] observed that anaerobic manure treatment of swine manure had an effect on particle size. The smallest particle fraction was between 2 and 10 μm for anaerobic digestates. This would suggest that a pretreatment for removing particles larger than 10 μm would be sufficient as a pretreatment for MF. However, Lee et al. [136] suggested that the smaller the particle size, the bigger the permeate flux decline because small colloids are more likely to aggregate, which would provoke an increase in cake resistance. During manure filtration, Fugère et al. [71] reported that the particle size of the treated manure could influence UF membrane fluxes and possibly define the membrane pore blocking mechanism and cake layer compactness.

### 6.4. Influence of Temperature

Temperature is an important parameter to take into account during evaluation of membrane performance. An increase in temperature produces a decrease in effluent viscosity and an increase in permeability [72]. This could imply lower membrane costs, a smaller membrane plant footprint and reduced operating costs. However, high temperatures, such as those for thermophilic anaerobic digestion (~55 °C), could also produce irreparable damage to polymeric membranes [6]. It is a good strategy thus to normalize the obtained permeate fluxes by correcting the experimental temperature using the manufacturer’s correction factor for the particular membrane [6].

During UF of anaerobically digestated swine manure liquid fraction, increasing the operation temperature decreased the performance and selectivity of PES external tubular membranes compared to PVDF submerged hollow fiber membranes. [72].

Masse and Massé [147] showed that the flocculation performance of high molecular weight cationic polymers during flocculation of high DM swine manure was not affected by the operating temperature (6–25 °C). Furthermore, there was no negative effect from temperature on the removal efficiency of TSS and phosphorus during the process. However, animal wastewater processing at high temperatures implies losing nitrogen due to ammonia volatilization [6]. In the same study, during RO processing of dairy and raw pig slurries, it was found that permeate flux increased by about 50% by increasing the operation temperature from 10 to 20 °C. However, when the volume reduction reached 60%, the flux decreased even though the temperature was still approximately 44 °C. This flux decrease was attributed to the rapid volume reduction with the related fast increase in osmotic pressure.

### 6.5. Influence of pH

Acidification is a common practice in agricultural manure management to reduce greenhouse gas emissions as well as to increase the fertilizer value of slurry [148]. The influence of the wastewater pH on membrane performance and compound rejection has been previously studied mainly for RO membranes. In general, small amounts of acid are recommended to avoid considerably altering the osmotic pressure of the wastewater for assisting MF-RO steps [21]. However, increases in pH in manure and digestate liquid fractions enhanced the removal of residual colloids [57].

During acidification of swine wastewater separation using RO membranes, it was found that acidification of the pretreated manure to a pH of 6.5, for typical swine manure containing about 3000 mg·L^−1^ of TAN, would ensure high TAN rejection ranging from 97.5% to 99.8% at a high permeate recovery rate of 80% [27]. However, when increasing the pH to 7 the permeate recovery rate had to be reduced to 50% in order to maintain the overall retention of TAN at 97% and concentrations of TAN below 100 mg·L^−1^ in the permeate. Additionally, a pretreatment of the swine wastewater with either NF or a first stage RO further increased feed pH values up to 8.5 due to a decreased alkalinity, which required less acid to lower pH but considerably reduced the TAN rejection [30]. Acidification also enhanced slightly K rejection on RO membranes, achieving a rejection of 71% in urea-source wastewater [115].

In another study, it was observed that during acidification, solubilization of P from the solid manure phase into the liquid phase might occur [148], promoting the dissolution of struvite. Christensen et al. [113] also suggested that pH adjustment could control the amount of dissolved phosphorus in manure. However, during micellar-enhanced UF (MEUF) applied for recovery of P, as pH increased, removal of heavy metals decreased to 80% due to P complex formation that was minimized at lower pH values [118].

### 6.6. Influence of Flocculants/Coagulants Used during Pretreatment

Physico-chemical methods alone, including coagulation-flocculation, have been found to be effective processes to remove solids and nutrients from animal manure [19,149]. The use of organic and inorganic coagulants and flocculants are recommended to achieve an optimal mechanical separation (e.g., centrifugation, gravitational sedimentation, flotation, gravity drainage, pressure filtration) between the obtained solid and liquid fractions. This results in a solid fraction containing mainly suspended solids, and a liquid fraction, containing most of the dissolved components originally in manure [5,99,150,151]. If nutrient recovery such as phosphorus is intended to be recovered from the obtained fractions to be re-used in agriculture, then Fe-containing coagulants or flocculants must be avoided. Other agents primarily used to precipitate dissolved phosphorus, such as salts of calcium, iron, or aluminum can also form precipitates with dissolved phosphorus. These agents also aid the coagulation/flocculation of suspended solids thus enhancing settling [70].

Electrostatic interactions due to the chemical structure of the flocculant are relevant in the subsequent membrane fouling mechanism. However, flocculation-coagulation can reduce membrane fouling at the optimal dosage by minimizing foulants attachment on the membrane surface due to the formation of larger flocs and restraining the formation of gel layer [152,153,154]. Furthermore, the addition of coagulants could decrease EPS and TOC concentrations in the effluent, thus minimizing membrane fouling [147]. For instance, membrane pressure control can be done with membrane assisted flocculation-coagulation techniques because total organic carbon (TOC) and EPS are major foulants that require a need of pressure increase [153,155].

Despite the benefits of flocculation-coagulation during manure separation, if dosing is not optimized, flocculation-coagulation could potentially cause severe membrane fouling [99,126]. The fouling capacity of polymer flocculants depends highly on the attraction-repulsion forces towards the membrane surface, flocculant molecular weight and solution chemistry (such as pH, solid concentration and presence of ions in the water solution) [99]. Additionally, pig manure contains highly charged particles suggesting that highly charged, high-molecular-weight cationic polymers can be used [113]. However, it was found that severe fouling could be caused by cationic flocculants, because of the electrostatic attraction with the negatively charged membrane surfaces [156]. Masse et al. [157] found that the amount of cationic polymer added during manure pretreatment for NF and RO membranes, should be closely related to the suspended solids present in solution, to minimize the residual polymer in solution. Residual polymer could play a detrimental role in membrane flux as it could facilitate rapid attachment of organic foulants on the membrane surface.

During the application of flocculants and coagulants on manure, Hjorth et al. [151] also observed that the polymer dose required to obtain large flocs during manure treatment was unaffected by the coagulant addition. It was observed that coagulation was minimal and did not significantly affect the polymer flocculation [158]. However coagulation can disturb the stability of colloidal organic matter, forming flocks by adsorption of dissolved materials and hence reducing the membrane fouling potential [152].

Zhu et al. [152] characterized membrane fouling on a MF ceramic membrane system treating secondary effluent. They showed that coagulation with Al was an effective pretreatment for the control of MF fouling with stable operating time and recovered water volume. However, it was suggested that the potential formation of larger complexes due to the presence of multivalent ions, such as Al and Ca, and organic molecules in the feed water, could also lead to the formation of gel layer or even membrane pore clogging. Similarly, Camilleri-Rumbau et al. [19] observed that digestate centrifugation assisted with flocculation-coagulation could alleviate membrane fouling, thus causing a positive impact on the long-term stability of the subsequent ultrafiltration step.

## 7. Membrane Cleaning and Ageing

Membranes processing highly charged wastewater effluents such as raw slurries or digestate manure require a frequent and efficient cleaning strategy in order to minimize the permeate production loss and restablish membrane performance. The fouling composition should dictate the cleaning strategy to be performed to maintain a stable membrane operation and ensure a long life-time operation. A proper understanding of membrane fouling could avoid excess of chemical cleanings, which increase operation times and cost. Therefore, it is important to determine the type of fouling interactions to establish better cleaning strategies [159].

A standard cleaning strategy takes into account the nature of the foulants in the wastewater and fouling mechanisms in order to select an optimal cleaning procedure. A cleaning agent can affect fouling in three ways (i) removal of the foulants, (ii) changing morphology of the foulants (i.e., swelling or compaction) and (iii) alteration of surface chemistry modifying membrane surface hydrophobicity or charge [159]. However, the achieved cleaning efficiency depends greatly on the nature of the foulant. Traditionally, membranes are cleaned with bases, acids, chelating agents, surfactants, salts, disinfectants, ozone, etc. in combination with deionized water flushing, high temperature, concentration of and contact time between cleaning agent and membrane [160]. Specifically, for RO and for FO, a limiting factor on the application of this membrane techniques is the precipitation of salts on the membrane surface or in the membrane structure, so-called scaling, occurring during water removal.

There are few studies where cleaning strategies are systematically studied on membranes processing farm effluents. For instance, Masse et al. [128] studied the cleaning efficiency of a combination of EDTA-SDS-NaOH solutions to recover flux and remove proteins and bacteria from RO membranes processing raw swine manure. They observed that the SDS-NaOH solution combination could successfully recover membrane flux, while the addition of EDTA did not improve flux. Additionally, flux recovery using NaOH alone could also be achieved by increasing the cleaning time (from 60 to 120 min) and pH (pH 11/40 °C and pH 12/33 °C). In line with these results, Camilleri-Rumbau et al. [92] further validated that NaOH alone could recover membrane flux succesffuly in long-term operations and that membrane flushing and soaking are important factors to preserve membrane stability. During UF of digested manure, alkaline and acidic cleaning cycles (NaOH and citric acid) were also effective in removing most of the inorganic foulants accumulated on the polysulfone membrane surface [19]. For UF-membranes cleaning and fouling have been reviewd by Shi et al. [161] to which interested readers are referred.

As stated above, temperature is also an important factor influencing foulant detachment of the membrane surface and structure. Madaeni et al. [162] found that, when cleaning RO membranes fouled with domestic wastewater, a flux recovery of 98% could be achieved by increasing the temperature of the cleaning solution to 35 °C, probably due to an increase in the transport rate and solubility of the cleaning agent and foulants [163]. Conversely, Masse et al. [128] found that the cleaning efficiency of the chemical solutions studied in their research increased significantly when pH was increased to 11–12, even if temperature had to be reduced to 35–40 °C.

Membrane rinsing or flushing with water has also been used successfully during cleaning of membranes fouled with farm effluents [115,127,139]. It was observed that membrane fouling resistance was reduced significantly after flushing [92,139]. All in all, membrane cleaning efficiency is a function of multiple parameters such as hydrodynamic conditions, concentration and temperature of chemical cleaning solution, as well as the order of the steps in the cleaning sequence. Some of the parameters, such as pH and temperature have strong non-linear effects on the cleaning effectiveness [164]. The required cleaning time is also a crucial parameter related to the cleaning efficiency. Generally, by increasing the cleaning time, the flux recovery increases sharply up to a certain limit. This means that the removal of loose deposits takes place during an optimal time after which the continuation of the cleaning process cannot significantly remove the strongly adsorbed fouling materials [126,128].

In addition to chemical cleaning methods, physical or mechanical membrane cleaning methods are also of interest, such as compressed air [165], air scrubbing [166] or ultrasound [167]. Backwashing can also be applied in ceramic membranes [151] and, with care, for polymeric MF and UF [97,166] membranes depending on the manufacturer’s recommendation. A more extended review of chemical and mechanical cleaning techniques is reported respectively in Sections S2 and S3 of the Supplementary Materials.

Ageing or membrane degradation has been studied previously in relation to membrane cleaning procedures. Former studies have investigated accelerated ageing protocols to assess the long-term impact of low concentrations of chemical compounds on membranes [168,169]. However, this strategy does not consider a realistic chemical cleaning duration compared to the operating conditions generally applied in the industry. Camilleri-Rumbau et al. [91,92] showed that the effect of the chemical traces even after membrane rinsing and a three-day soaking in permeate water further improved the membrane flux recovery, without apparent increase in membrane degradation during the processing period considered. During cleaning of NF membranes fouled by conventionally-treated surface water, Liikanen et al. [170] found that the ion retention loss was most profound in the cleanings containing only alkaline chelating cleaning agents, while acidic cleaning agents played an important role in preserving membrane ion retention. However, other studies showed that the concentration of the feed solution has the highest contribution in ion rejection, whilst transmembrane pressure and temperature of feed solution have minor contributions [171].

During cleaning, membrane modification can also occur. Nyström and Zhu [172] suggested that cleaning may initially increase the membrane flux partly by ridding the pores of the material that is left from the membrane preparation process, and partly by making the pore surfaces more hydrophilic and charged by the adsorption of the cleaning agent. The increased hydrophilicity makes the chemical bonds between the water molecules and surface groups of the membrane stronger, thus reducing the possibilities of the foulants to displace water molecules and adhere on the membrane. Modification of the membrane can also significantly reduce the contact angles measured. Lower contact angles indicate more hydrophilic membranes and such membranes will potentially show better resistance to fouling by hydrophobic foulants [32]. An increased charge of the membrane increases the electrostatic repulsion between the active sites of the membrane, and thus makes the membrane more open [173]. An increased charge of the membrane also increases the repulsive forces between the membrane and similarly charged foulants. In addition to the increased flux, the membrane modification after cleaning cycles can result in lower ion retention, although this retention is often recovered during membrane operation [174].

## 8. Environmental Advantages of Membrane Separation Processes

The main advantage of incorporating membrane unit operations in farm effluents processing is that the high-quality membrane permeates are particle- and pathogen-free regardless of the initial quality of the feedstock. This allows for nutrient recovery in the form of solid and liquid fertilizers, and water recycling. The separation itself is a physical procedure that does not require use of chemicals other than the cleaning agents. Furthermore, due to their modular nature, membrane units are packed in compact systems with lower footprint that are easily scalable.

From a life cycle assessment perspective, the environmental implications of membrane technology transcend the target separation application, and does also include factors such as membrane manufacturing, transportation, operation, cleaning, reuse and ultimately membrane end-of-life management [175,176].

Tangsukbul et al. [175] performed life cycle assessment of wastewater treatment by microfiltration at different operating conditions. In general, they observed that the highest contribution to environmental impact was that of the energy consumption, followed by membrane manufacturing, chemical usage, and ultimately transportation, which was not significant. Most of the energy demand in membrane processes is attributed to pumping, due to the need of high shear and cross-flow velocities in order to minimize fouling. Different approaches have been reported to reduce energy demand in membrane processes. Tangsukbul et al. [175] recommended operating at relatively low fluxes to reduce energy consumption, however more membrane modules would be needed to achieve the same production, and that would imply moving the problem elsewhere. In this regard, operation at a competitive flux regime however using renewable energy sources was recommended as the option most benign to the environment. With a completely different approach, Gienau et al. [177] reported that modifying the viscosity and rheological behaviour of the feedstock by means of enzymatic pre-treatment could save up to 45% of the energy demand during ultrafiltration of manure digestate keeping the same flow conditions.

Due to fouling and ageing, the membranes need to be cleaned regularly and substituted periodically. Thus, the lifespan of a membrane is directly affected by the nature and severity of the membrane fouling. When membrane performance decays below an acceptable level, the modules are disposed as waste, mostly landfilled. Landarburu-Aguirre et al. [176] reviewed different end-of-life strategies for desalination RO modules including strategies for reuse and recycling. For instance, in some cases the membranes could be cleaned and re-used as RO membranes in processes requiring less water quality, or as NF or UF membranes [178]. However, reusing highly fouled membranes used for animal effluent treatment may be challenging due to the complexity, heterogeneity and possibly pathogenic nature of the foulants. Especially for the membranes used in the initial stages of the process, use of disinfectants would be indispensable prior to eventual membrane recycling. In the particular application of farm effluent processing, adequate fouling prevention and mitigation seems the most adequate approach towards extending membrane lifetime. The environmental impact of membrane cleaning in terms of type of cleaning agent, chemical concentration and disposal also need to be taken into consideration in the environmental assessment of a given process. In a more general perspective, Nunes et al. [179] outline the challenges and prospects in the future of membrane technology, where innovation and sustainability play key roles on the development and manufacturing of novel membranes and in design of separation processes for a sustainable future.

All in all, the implementation of membrane processes can undoubtedly help reduce the environmental burdens associated to animal waste processing and turn the waste into a valuable resource.

## 9. Future Research Needs

Membrane technologies offer the potential to separate and concentrate the fertilizers present in animal livestock effluents while at the same time producing clean effluents that can be discharged to the environment. The effective usage of membrane technologies in this respect is challenged by (a) the separation of fertilizers from heavy metals, (b) membrane fouling and the need for fouling treatment and (c) the need of a sustainable production, usage and disposal of membranes.

Phosphates and heavy metals are mainly associated with particulate matter in the livestock effluents and the separation of these, apart from the membrane molecular weight cut-off, is influenced by the membrane fouling layer. A better understanding of fouling layer built-up during operation is needed to address points (a) and (b) above. Most methods used to characterize membrane fouling are destructive methods not allowing real-time three-dimensional characterization of the fouling built-up, nor a true picture of how the fouling layer is influenced by fluid dynamics in the membrane module. A relatively novel technique, X-ray tomographic microscopy soft tissue in situ imaging, might just offer that option [180]. This would also help targeting another issue with fouling, a proper treatment and removal of the fouling layer. As most chemicals used for removal of fouling are not environmental-friendly, a more precise targeting and reduced usage is wanted, and alternative green cleaning agents must be developed.

The need for a sustainable production, usage and disposal of membranes is well recognized [179]. Recycling membranes used in treatment of biological waste streams might be difficult due to the risk of pathogens. Controlled chemical modification through oxidation of polyamide active layers could be an alternative for giving a second life to the resulting converted membranes [181].

Considering the short life span for membranes treating livestock effluents, developing low-cost disposable biodegradable membranes based on biopolymers [182] should be a future must for making membrane technology a viable solution in livestock effluent treatment.

## 10. Conclusions

Membrane fouling represents one of the major drawbacks when using membrane technologies during farm effluent processing. However, by understanding the related mechanisms, fouling composition and establishing efficient membrane pretreatment and cleaning strategies, membrane technologies can lead to an outstanding performance in terms of volume reduction and nutrient recovery. This paper reviews the raw and digestate general physico-chemical characteristics, technologies used for solid-liquid separation pretreatment at farm level, membrane characteristics requirements for treating liquid fractions of raw and digested manure, as well as membrane fouling mechanisms and membrane cleaning strategies described in the literature. Additionally, this review paper also provides valuable information about recovery of nutrients from manure, such as ammonia-nitrogen and phosphorus as well as heavy metal removal from these effluents using membranes.

## Figures and Tables

**Table 1:** Main manure SLS systems with characteristics of obtained liquid fractions.

Solid-Liquid Separation (SLS) System	Characteristics	Type of Feed	DM [%]		Reference
			Feed	Obtained Liquid Fraction after SLS	
**Decanter centrifuge**	Alfa Laval, NX 309B-31, Denmark)	Pig manure co-digestate	4.8 ± 0.2	2.1 ± 0.09	[66]
	AD-1220, GEA, Germany	Pig, cattle and chicken manure co-digestate	NA	2.7 (MF feed)	[26]
	Gennaretti centrifuge GHT VF, Italy	Pig slurry co-digestate	4.56	2.2	[19,68]
	Centrifuge, Bauer GmbH	Cow co-digestate	NA	4	[20]
**Screw press**	FAN separator, max. feed rate 6.5 m^3^·h^−1^	Sow slurry	1.5–2	NA	[21]
	NA	Dairy manure digestate	NA	4.1 ± 0.3	[69]
**Filter press**	ID construct (chamber filter press), 0.20–0.35 m^3^·h^−1^, 10–15 bar, polyamide-based filter cloth Rilsan^®^ types R43 and R57	Sow slurry	1.5–2	NA	[21]
**Vibration screen**	CRETEL, max. feed rate 1 m^3^·h^−1^, 0.5 mm mesh size	Sow slurry	1.5–2	NA	[21]
**Liquid cyclone**	SRC, 15 m^3^·h^−1^, 5–7 bar, diameters of 7,12 and 18 mm	Sow slurry	1.5–2	NA	[21]
**Bag filter**	800 μm mesh size	Sow slurry	1.5–2	NA	[21]
**Tangential flow separation**	System supplied by Gaston County Dyeing Machine Company (GCDMC)	Pig manure (flushed waste)	1.11 ± 0.49	0.96 ± 0.31	[70]
**Sedimentation tank**	Settling basin	Sow slurry	1.5–2	NA	[21]
	Storage tank	Pig manure	2.0	NA	[71]
**Combined: screw press + centrifugation + MF**	Screw press: FAN Separator, pore diameter of 0.25 mm; decanter centrifuge: Baby II; capacity of 0.7 m^3^·h^−1^; 7.5 kW (Pieralisi, Italy); MF pore size 0.2 μm (Zenon GmbH, Germany)	Liquid pig manure	5.1 ± 2.2	8.1 ± 1.0 (MF concentrate)	[60]
**Combined: straw filter + sedimentation**	Uncompressed straw bed on a trenched concrete floor and settling tanks	Liquid pig manure	6.1 ± 1.1	1.9 ± 0	[60]
**Combined: nitrification-denitrification + sedimentation**	Nitrification/denitrification system and settling basin	Sow liquid manure	5.3 ± 2.9	0.91 ± 0.04	[58]

Values are expressed as average ± standard deviation.

**Table 2:** Combined manure treatment systems.

Combined Manure Treatment Systems	Operation Units	Reference
	Ceramic MF membranes and polymeric RO membranes membrane technologies. RO was fed with the microfiltrate in sow slurry separation to obtain a high-quality liquid fraction	[21]
**PIGMAN concept**	Decanter separator, stirred tank and up flow anaerobic sludge blanket reactors, post digestion, partial oxidation and oxygen-limited autotrophic (nitrification-denitrification) (OLAND)	[66]
**AnMBR + UF**	Anaerobic membrane bioreactor with ultrafiltration	[72]
**Several case studies on digestate post-treatment technologies**	AD, ammonia stripping and membrane separation based on MF, NF and RO	[73]
**BIOREK^®^ concept**	AD, ammonia stripping and membrane separation based on UF and RO	[74]
**ADEPT (AD Elutriated Phased Treatment) -SHARON (Single reactor system High Ammonium Removal Over Nitrite)-ANAMMOX (Anaerobic Ammonium Oxidation)**	hydrolysis/acidification reactor, methanogenic reactor, SHARON-ANAMOX reactor	[75]
**Several case studies on manure treatment technologies**	Combined, among other techniques, AD, centrifuge separation with flocculation, acidification, nitrification and de-nitrification, combination of anaerobic digestion–evaporation and drying composting, etc.	[76]

## Supplementary Materials

The following are available online at https://www.mdpi.com/1660-4601/18/6/3107/s1, Table S1: Composition of different raw manure, Table S2: Classification of membrane-foulant characterization technique, Section S1: Techniques for fouling and membrane characterization, Section S2: Chemical cleaning, Section S3: Mechanical cleaning.
