# Supplementary material for: Treatment of Manure and Digestate Liquid Fractions Using Membranes: Opportunities and Challenges
Source: Int J Environ Res Public Health. 2021 Mar 17;18(6):3107. doi: 10.3390/ijerph18063107 (PMC8002686; doi:10.3390/ijerph18063107)
Supplement: Supplementary file 1 [file ijerph-18-03107-s001.pdf]

International Journal of
Environmental Research and Public Health

MDPI

Review – Supplementary Material

Treatment of manure and digestate liquid fractions using membranes: Opportunities and challenges

Maria Salud Camilleri-Rumbau*[1,2], Kelly Briceño[1], Lene Fjerbæk Søtoft[1], Knud Villy Christensen[1], Maria Cinta Roda-Serrat[1], Massimiliano Errico[1] and Birgir Norddahl[1]

[1] University of Southern Denmark, Faculty of Engineering, Department of Green Technology, Campusvej 55, 5230 Odense M, Denmark
[2] Aquaporin A/S, Nymøllevej 78, 2800 Kongens Lyngby, Denmark

* Correspondence: macarum@gmail.com

Content

Table S1: Composition of different raw manure
S1. Techniques for fouling and membrane characterization
Table S2: Classification of membrane-foulant characterization techniques (M: membrane; F: foulant).
S2. Chemical cleaning
S2.1 Alkalis
S2.2 Surfactants
S2.3 Chelating agents
S2.4 Acids
S2.5 Disinfection
S2.6 Ozonation
S3. Mechanical cleaning
References

Table S1. Composition of different raw manure.

Effluent	pH	TVS (g·kg^{-1})	TS (g·kg^{-1})	DM (g·L^{-1})	TKN (g·kg^{-1})	TAN (g·kg^{-1})	P (g·kg^{-1})	K (g·kg^{-1})	Reference
Cattle manure	7.4	-	-	63.7	2.5	1.7	0.69	3.54	[1]
	NA	-	-	82 ± 24	-	5.0	1.0 ± 0.2	4.3 ± 1.8	[2]
	7.3	85 ± 0.03	115 ± 4.5	-	4.6 ± 0.06	-	-	-	[3]
Poultry manure	-	-	277.18 ± 20.24	NA	13.09 ± 4.16	-	4.73 ± 0.91	23.07 ± 1.27	[4]
Horse manure	7.9 ± 0.1	-	-	-	-	-	5.2 ± 0.4	20.8 ± 1.2	[5]

S1. Techniques for fouling and membrane characterization

Different characterization techniques have been reported as a tool for investigating the membrane fouling phenomena (Table S2). The purpose of this section is to highlight the main characterization techniques applied during practical evaluation of full-scale membrane plants. The proposed analytical tools are divided in membrane and foulant analysis.

Table 2. Classification of membrane-foulant characterization techniques (M: membrane; F: foulant).

Membrane Analysis	Output	Application	Analysis of:		Reference
			M	F	
Visual Inspection	Material damage, thin film layer delamination	UF: broken fibers are analyzed after module inspection, fibers are often preserved in MBS at least any further organic characterization would be requested<br>RO and NF: Fujiwara test is used to determine the integrity of the polyamide layer after exposure to oxidizing halogens such as chlorine<br>Rhodamine B test also exposes the support as a consequence of PA layer delamination after operation or intensive cycles of cleaning.	X	X	[6]
SEM	Morphology, thickness, fouling structure, pore size distribution	Biofouling and scaling can be inspected easily<br>Sample preparation includes sample preparation using liquid N2 and metallization.	X	X	[7]
AFM	Roughness, porosity, surface profile	Useful tool to analyze the impact of different cleaning strategies in order to optimize the cleaning protocols	X		[8]
EDX	Inorganic analysis	Brings information about membrane scaling, membrane degradation	X	X	[9]
XRD	Structure of inorganic substances	Allows determining whether the presence of particles in the fouling layer has an amorphous or crystalline structure as in the case of the presence of halite (NaCl) and hydrogen or alum inosilicates, H(AlSi2O5)		X	[10]
FTIR-ATR	Organic groups, Polyamide integrity	Determine the presence of specific functional organic groups that may contribute to fouling layer formation. Useful to determine the nature of the organic components based on proteins, polysaccharides and aromatic compounds that can be derived from humic and fulmic acids.	X	X	[11,12]
Streaming potential	Membrane and foulant charge	Brings information about membrane surface charges and the type of foulants that are in contact with them. E.g., humic acids are expected to make the membrane more negative while proteins favor positive charges	X	X	[13-16]
Contact angle	Hydrophilicity character of the membranes	Can be considered to explain the fouling trends in a polymeric material. The general approach includes the measurement of the angle between a droplet of water and the membrane surface.	X		[9,17]
Pyrolysis GC-MS	Characterization of natural organic matter	Useful to determine the presence of biopolymers in organic matter. The pyrograms bring information about polysaccharides, proteins, aminosugars, polyhydroxybutyrates, deoxyribonucleic acid (DNA), lipids/fatty acid and polyhydroxyaromatic compounds.	X	X	[18]
ICP-OES	Quantitative analysis of the elements constituting the deposits	ICP-OES can be used to identify both organic and inorganic elemental constituents in foulants and when it is combined with loss on ignition test it is possible to determine the relative percentages by weight of the organic and inorganic fraction of foulants.	X	X	[19]
IC	Analysis of Ions	Ion Chromatography. This technique provides valuable quantitative results concerning the main water cations and anions		X	[19]
AES	Ion concentration	Atomic emission spectrometry. Ion concentration determination		X	
SEC	Concentration of biopolymers	Size Exclusion Chromatography. This technique is useful for the determination of dissolved organic matter (DOM) rejection. It can be integrated with online dissolved organic carbon (DOC) and UV detection at 254nm (LC-DOC) [72] and apparent molecular weight distribution (AMWD) of organic substances		X	[20,21]
Potentiometric titration methods	Interaction foulants /membrane	Used for determination of carboxylic and phenolic acidity.		X	[17]
ATP (adenosine triphosphate	Biological activity	Portable devices offer a fast alternative to detect biological activity in the evaluation of UF cleaning efficiency, or plant sanitization. Biofouling can be assessed after analysis of ATP.		X	[18]

S2. Chemical cleaning

S2.1. Alkalis

Alkaline solutions, such as NaOH, at high concentrations and temperatures are capable of cleaning organic-fouled membranes by increasing the negative charges, causing first hydrolysis and later solubilization of proteins or causing saponification and dissolution of proteins [22,23]. Alkaline cleaning environments and removal of foulants from the membrane structure can lead to membrane charge modifications making the membrane

more open, obtaining thus a considerable flux improvement after cleaning [24]. Beyer et al. [25] found that, although a lower permeate flux recovery was obtained with a NaOH solution (pH 11) in the absence of SDS, the permeate flux after cleaning was stable. Zhang and Liu [26] observed that, although a HCl aqueous solution cleaning step reduced the membrane flux severely due to a dense gel layer formation, a subsequent cleaning with NaOH aqueous solution allowed the flux to increase immediately.

S2.2. Surfactants

When using anionic surfactants, such as SDS, Beyer et al. [25] demonstrated that the disruption of the organic–calcium complex by SDS at pH 11, resulted in a cleaning efficiency of more than 100% flux. This complex caused a dense and compacted fouling layer on NF membranes fouled with proteins in simulated secondary treated effluent. The explanation is that SDS can effectively break down the organic gel network by disrupting the complex between the organic foulant and divalent cations such as Ca. Chen et al [27] suggested that the application of anionic surfactants appeared to modify the contribution of the charge effects to the long term fouling process during BSA passive adsorption on membranes pretreated with surfactants. Other studies showed also that a mixture of SDS using NaOH could lead to permeate flux recoveries of around 95% [28-30]. Most of the afore-mentioned research on fouling intensity and cleaning were conducted on laboratory-scale unit with [31]. When fouling is formed on the membrane surface, an efficient cleaning can be achieved by considering the chemical reaction, mass transfer and the optimization of cleaning conditions [32,33].

The higher cleaning efficiency achieved when using surfactants could be partly due to the adsorption of surfactants in hydrophobic areas, which are potential protein adsorption sites. Surfactants and alkaline solutions could also provide a more negatively charged surface that repeal proteins. In this regard, during pre-cleaning of NF membranes with Ultrasil 10 solution, it was found that membrane hydrophobicity increased according to the contact angle measurements. This might cause more hydrophobic interactions after cleaning, that might lead to flux decline [34]. In addition, the presence of added anionic species may orient the proteins on the membrane surface for favorable electrostatics, resulting in a less optimal conformation for hydrophobic interactions [27,35].

S2.3. Chelating agents

The addition of metal chelating agents into the chemical cleaning solution, such as EDTA, caused no significant flux recovery [26] effect on RO membranes processing swine wastewater [36-38]. Additionally, Zhang et al. [39] found that cleaning with EDTA at pH 10 and NaOH at pH 2 at 25˚C resulted only in a limited further flux recovery. However, the introduction of alkaline chelating agent (Na_4EDTA) increased the membrane permeability more than plain alkaline cleaning (NaOH), during NF of conventionally-treated surface water [24], probably by complexing some constituents from the membrane structure, making the membrane more open and permeable.

Additionally, in the presence of divalent Ca ions, organic molecules with carboxylic groups tend to form metal-organic complexes. Previous studies showed that the addition of calcium to a secondary effluent organic matter at pH 7 increased pressure. One possible reason for this is the increase in proton H^+ concentration, which inhibits the protolysis of carboxylic groups and divalent cations, preventing the formation of bridges between them [21,40].

S2.4. Acids

Acidic cleaning showed to have limited flux recovery compared to alkaline cleanings [26,30,36]. This could be related to the membrane charge increasing in an alkaline environment, provoking a more open membrane and an increase in the repulsive charges between the membrane and the charged foulants [24,27]. Masse et al. [36] found that small reductions in flux were observed when RO compacted virgin membranes were cleaned with citric acid alone or after alkaline cleaning. Additionally, the use of HCl and HNO_3 as

a first cleaning step was detrimental [28]. In this regard, Zhang et al. [39] found that, cleaning digestate-fouled polyethersulphone ultrafiltration membranes with 0.1.N HNO_3 at different temperatures (25, 37 and 50°C) achieved a complete removal of inorganic precipitates but no flux recovery. However, a citric acid cleaning step showed an improved removal of inorganic elements compared to NaOCl during cleaning of MF ceramic membranes treating secondary effluent [20]. As reviewed by Porcelli and Judd [41], whilst strong mineral acids can solubilise deposits, organic acids, such as citric and oxalic, are more effective in capturing organo-metallic foulants acting as a chelating agent and providing buffering abilities with the advantage of lowering the risk of damaging the membranes.

S2.5. Disinfection

Disinfectants such as chlorine can be used during membrane biofouling cleaning. However, chlorine is not recommended for cleaning polymeric membranes [22,42] unless used at low concentrations and short periods. Saravia et al. [21] used deionized water with NaOH (pH value at about 10) or NaClO (200 ppm) solution to clean MF-UF submerged hollow fiber membrane module fouled with different concentrations of dissolved organic matter water and calcium ions during filtration of water with high concentration of dissolved organic carbon investigated using NaCl and $CaCl_2$ $2H_2O$. After the cleaning procedures the initial flux could be recovered. Boerlage et al. [43] also found that almost 100% of the initial water flux of PAN membranes could be recovered with just one cleaning with NaOCl when fouled with tap water. In contrast, PS membranes needed several chemical cleanings.

Although chlorine cleaning is not recommended by some manufacturers due to damaging risk of the polyamide surface [44] due to membrane degradation which can lead to a decrease in salt rejection [6], mostly related to the formation of ring chlorinated products under high pressure. Salt solution such as NaCl was the least efficient cleaning strategy to clean membranes fouled with swine wastewater [36]. However, the use of NaOCl and HCl, had positive effects on the flux recovery achieved by RO membranes processing swine wastewater [36]. Low concentrations of NaOCl were also able to remove particles from membrane surface and pores due to its strong oxidizing potential in PS membranes fouled with milk components. This was due to enhanced hydrophilicity of membrane surface that lead to permeate flux increase.

S2.6. Ozonation

Ultimately, if sanitation needs to be achieved, ozonation for handling micropollutants on the wastewater stream can also be applied [45]. Ozonation reduces the organic content by partial mineralization and degradation of larger molecules. Moreover, by reducing the size of the organic molecules and mineralizing part of them, the resulting smaller molecules can pass through the membrane pores, being less likely to be captured and more prone to the formation of a loose and thin gel layer [21,40]. Ozonation was also used on during RO pretreatment. However, together with acidification, these pretreatments did not reduce the particulate fouling potential successfully. Antiscalant addition did also contribute to add particles to RO feed water but in a less extent than adding acid. However, activated carbon and slow sand filtration (SSF) did help significantly in removing particles from the feed water [43]. Pre-ozonation of the organic matter of secondary effluents and its fractions reduced membrane fouling during MF [17]. It was observed that pre-ozonation changed the gel layer from a hydrophobic surface into a hydrophilic one. Carboxylic and phenolic groups slightly increased also after ozonation, reducing pH. This change might also enhance membrane organic fouling under the presence of multivalent ions such as Ca.

S3. Mechanical cleaning

Mechanical cleaning can be used as a first step during membrane cleaning. The residual cleaning that cannot be eliminated by this strategy must be removed by chemical

cleaning. The complexity of mechanical cleaning can range from a simple membrane cleaning procedure can include a physical scrubbing of the membrane surface by a sponge ball [46] to more complex cleaning methods which imply intermittent forward flushing with water [17], air and liquid backwashing [20,21,43] (in ceramic membranes and sometimes in MF and UF polymeric membranes), air scouring [21], ultrasounds [46], etc.

Studies showed that gas bubbling membrane cleaning of MF flat sheet membranes lead to an increase of 20% in permeate productivity when applying flux intermittently and air bubbling was introduced in the zero flux periods. The limitations of introducing air scouring and high-pressure air in a system are mainly high operation costs via energy usage and initial investment costs. Other problems that could rise with air scouring is foaming [48].

Especially backwashing is of great importance in removing internal fouling, although in some cases it failed to completely remove the accumulated particles [48]. Suspended solids in feed water were the main cause of reversible fouling which was controlled during the backwashing cycles [20].

Sonification (at 42kHz and 20˚C) is also a mechanical membrane cleaning strategy which can help to minimize irreversible fouling to < 5% of the flux reduction. Ultrasound cleaning was also applied in studies based on BSA fouling [48] and glutamate processing wastewater with consideration of Fe, Ca and Mg [49]. Other studies using vibratory shear enhanced processing membrane filtration showed that during MF and UF this system could operate efficiently even when applying high trans-membrane pressures that enhance membrane fouling [50]. The same concept has also been applied to reverse osmosis membranes, known as VSEP® [51].

Turbulence promoters of mesh feed spacers were also used to create hydrodynamic flow conditions helping during control of foulant deposition [52].

References

Møller, H.B.; Sommer, S.G.; Ahring, B.K. Separation efficiency and particle size distribution in relation to manure type and storage conditions. Bioresource Technology **2002**, *85*, 189-196.

Hjorth, M.; Christensen, K.V.; Christensen, M.L.; Sommer, S.G. Solid-liquid separation of animal slurry in theory and practice. A review. Agron. Sustain. Dev. **2010**, *30*, 153-180.

Hartmann, H.; Ahring, B. Anaerobic digestion of the organic fraction of municipal waste: Influence of co-digestion with manure. Water Research **2005**, *39*, 1543-1552.

Borowski, S.; Domanski, J.; Weatherley, L. Anaerobic co-digestion of swine and poultry manure with municipal sewage sludge. Waste Manag. **2014**, *34*, 513-521.

Hanc, A.; Enev, V.; Hrebeckova, T.; Klucakova, M.; Pekar, M. Characterization of humic acids in a continuous-feeding vermicomposting system with horse manure. Waste Management **2019**, *99*, 1-11.

Antony, A.; Fudianto, R.; Cox, S.; Leslie, G. Assessing the oxidative degradation of polyamide reverse osmosis membrane-Accelerated ageing with hypochlorite exposure. J of Membrane Science **2010**, *347*, 159-164.

Tylkowski, B.; Tsibranska, I. Overview of main techniques used for membrane characterization. Journal of Chemical Technology and Metallurgy **2015**, *50*, 3-12.

Espinasse, B.P.; Chae, S.-R.; Marconnet, C.; Coulombel, C.; Mizutani, C.; Djafer, M.; Heim, V.; Wiesner, M.R. Comparison of chemical cleaning reagents and characterization of foulants of nanofiltration membranes used in surface water treatment. Desalination **2012**, *296*, 1-6.

Zabreska, A.; Romero Nieto, D.; Christensen, K.V.; Norddahl, B. Ammonia recovery from agricultural wastes by membrane distillation: Fouling characterization and mechanism. Water research **2014**, *56*, 1-10.

Melian-Martel, N.; Sadhwani, J.J.; Malamis, S.; Ochsenkuhn-Petropoulou, M. Structural and chemical characterization of long-term reverse osmosis membrane fouling in a full scale desalination plan. Desalination **2012**, *305*, 44-53.

Zhu, H.; Nystrom, M. Cleaning results characterized by flux, streaming potential and FTIR measurements. Colloids and Surfaces A: Physicochemical and Engineering Aspects **1998**, *138*, 309-321.

Shon, H.K.; Vigneswaran, S.; Kim, In S.; Cho, J.; Ngo, H.H. Fouling of ultrafiltration membrane by effluent organic matter: A detailed characterization using different organic fractions in wastewater. J of Membrane Science **2006**, *278*, 232-238.

Camilleri-Rumbau, M.S.; Norddahl, B.; Wei, J.; Christensen, K.V.; Søtoft, L.F. Microfiltration and ultrafiltration as a post-treatment of biogas plant digestates for producing concentrated fertilizers. Desalin. Water Treat. **2014**, *55*, 1639-1653.

Nystrom, M.; Lindstrom, M.; Matthiasson, E. Streaming potential as a tool in the characterization of ultrafiltration membranes. Colloids and Surfaces **1989**, *36*, 297-312.

Le Bolay, N.; Ricard, A. Streaming potential in membrane processes: Microfiltration of egg proteins. J of Colloids and Interface Science **1995**, *170*, 154-160.

Lawrence, N.D.; Perera, J.M.; Iyer, M.; Hickey, M.; Stevens, G. The use of streaming potential measurements to study the fouling and cleaning of ultrafiltration membranes. Separation and Purification Technology **2006**, *48*, 106-112.

Zhu, H.; Wen, X.; Huang, X. Membrane organic fouling and the effect of pre-ozonation in microfiltration of secondary effluent organic matter. J Membrane Science **2010**, *352*, 213-221.

Khan, M.T.; Busch, M.; Molina, V.G.; Emwas, A.-H.; Aubry, C.; Croue, J.-P. How different is the composition of the fouling layer of wastewater reuse and seawater desalination RO membranes? Water Res. **2014**, *59*, 271-282.

Chen, S.C.; Amy, G.L.; Chung, T.-S. Membrane fouling and anti-fouling strategies using RO retentate from a municipal water recycling plant as the feed for osmotic power generation. Water Res. **2016**, *88*, 144-155.

Zhu, H.; Wen, X.; Huang, X. Characterization of a membrane fouling in a microfiltration ceramic membrane system treating secondary effluent. Desalination **2012**, *284*, 324-331.

Saravia, F.; Zwiener, C.; Frimmel, F.H. Interactions between membrane surface, dissolved organic substances and ions in submerged membrane filtration. Desalination **2006**, *192*, 280-287.

Cheryan, M. Ultrafiltration and Microfiltration Handbook. Second Edition, CRC Press 1998.

Mc Donnell, G.; Hansen, J. Block´s Disinfection, Sterilization, and Preservation. Sixth Edition, Wolters Kluwer, 2020.

Liikanen, R.; Yli-Kuivila, J.; Laukkanen, R. Efficiency of various chemical cleanings for nanofiltration membrane fouled by conventionally-treated surface water. J of Membrane Science **2002**, *195*, 265-276.

Beyer, M.; Lohrengel, B.; Nghiem, L.D. Membrane fouling and chemical cleaning in water recycling applications. Desalination **2010**, *250*, 977-981.

Zhang, G.; Liu, Z. Membrane fouling and cleaning in ultrafiltration of wastewater from banknote printing works. J of Membrane Science **2003**, *211*, 235-249.

Chen, V.; Fane, A.G.; Fell, C.J.D. The use of anionic surfactants for reducing fouling of ultrafiltration membranes: Their effects and optimization. J of Membrane Science **1992**, *67*, 249-261.

Mohammadi, T.; Madaeni, S.S.; Moghadam, M.K. Investigation of membrane fouling. Desalination **2003**, *153*, 155-160.

Ang, W.S.; Tiraferri, A.; Chen, K.L.; Elimelech, M. Fouling and cleaning of RO membranes fouled by mixtures of organic foulants simulating wastewater effluent. J of Membrane Science **2011**, *376*, 196-206.

Hijnen, W.A.M.; Castillo, C.; Brouwer-Hanzens, A.H.; Harmsen, D.J.H.; Cornelissen, E.R.; van der Kooij, D. Quantitative assessment of the efficacy of spiral-wound membrane cleaning procedures to remove biofilms. Water Research **2012**, *46*, 6369-6381.

Zhang, R.H.; Yang, P.; Pan, Z.; Wolf, T.D.; Turnbull, J.H. Treatment of swine wastewater with biological conversion, filtration, and reverse osmosis: A laboratory study. Transactions of the ASAE **2004**, *47*, 243-250.

Chen, J.P.; Kim, S.L.; Ting, Y.P. Optimization of membrane physical and chemical cleaning by a statistically designed approach. J of Membrane Science **2003**, *219*, 27-45.

Al Amoudi, A.; Lovitt, R.W. Fouling strategies and the cleaning system of NF membranes and factors affecting cleaning efficiency. J of Membrane Science **2007**, *303*, 4-28.

Liikanen, R.; Kiuru, H.; Tuhkanen, T.; Nystrom, M. Nanofiltration membrane fouling by conventionally treated surface water. Water Supply **2003**, *3*, 183-190.

Watanabe, N.; Shirakawa, T.; Iwahashi, T.; Seimiya, T. Effect of surface charge on adsorption of bovine serum albumin 2. Interaction of protein serum molecules with an anionic monolayer, as studied by ellpsometry, radiotracer and surface tension measurements. Colloid and Polymer Science **1988**, *266*, 254-260.

Masse, L.; Mondor, M.; Puig-Bargues, J.; Deschenes, L.; Talbot, G. The efficiency of various chemical solutions to clean reverse osmosis membranes processing swine wastewater. Water Quality Research Journal **2014**, *49*, 295-306.

Masse, L.; Puig-Bargues, J.; Mondor, M.; Deschenes, L.; Talbot, G. Efficiency of EDTA, SDS, and NaOH solutions to clean RO membranes processing swine wastewater. Separation Science and Technology **2015**, *50*, 2509-2517.

Masse, L.; Puig-Bargues, J.; Mondor, M.; Deshenes, L.; Talbot, G. The efficiency of EDTA+SDS solutions to clean RO membranes processing swine wastewater. 7th IWA Specialised Conference and Exhibition on Membrane Technology in Water and Wastewater Treatment, Toronto 2013.

Zhang, J.; Padmasiri, S.I.; Fich, M.; Norddahl, B.; Morgenroth, E. Influence of cleaning frequency and membrane history on fouling in an anaerobic membrane bioreactor. Desalination **2007**, *207*, 153-166.

Boerlage, S.F.E.; Kennedy, M.D.; Aniye, M.P.; Abogrean, E.M.; El-Hodali, D.E.Y.; Tarawneh, Z.S.; Schippers, J.C. Modified fouling $index_{ultrafiltration}$ to compare pretreatment processes of reverse osmosis feedwater. Desalination **2000**, *131*, 201-214.

N. Porcelli, S. Judd, Chemical cleaning of potable water membranes: A review, Separation and Purification Technology **2010**, *71*, 137-143.

Liikanen, R. Nanofiltration as a refining phase in surface water treatment. PhD thesis, Helsinki University of Technology 2006.

Boerlage, S.F.E.; Kennedy, M.D.; Dickson, M.R.; El-Hodali, D.E.Y.; Schippers, J.C. The modified fouling index using ultrafiltration membranes (MFI-UF): Characterization, filtration mechanisms and proposed reference membrane. J of Membrane Science **2002**, *197*, 1-21.

Buch, P.R.; Mohan, D.J.; Reddy, A.V.R. Preparation, characterization and chlorine stability of aromatic-cycloaliphatic polyamide thin film composite membranes. J of Membrane Science **2008**, *309*, 36-44.

Maurer, M.; Pronk, W.; Larsen, T.A. Treatment process for source-separated urine. Water Research **2006**, *40*, 3151-3166.

Fugere, R.; Mameri, N.; Gallot, J.E.; Comeau, Y. Treatment of pig farm effluents by ultrafiltration. Journal of Membrane Science **2005**, *255*, 225-231.

Lujan-Facundo, M.J.; Mendoza-Roca, J.A.; Cuartas-Uribe, B.; Alvarez-Blanco, S. Ultrasonic cleaning of ultrafiltration membranes fouled with BSA solution. Separation and Purification Technology **2013**, *120*, 275-281.

Karakashev, D.; Schmidt, J.E.; Angelidaki, I. Innovative process scheme for removal of organic matter, phosphorus and nitrogen from pig manure. Water Research **2008**, *42*, 4083-4090.

Wang, Q.; Yang, P.; Cong, W. Cation-exchange membrane fouling and cleaning in bipolar membrane electrodialysis of industrial glutamate production wastewater. Separation ad Purification Technology **2011**, *79*, 103-113.

Petala, M.D.; Zouboulis, A.I. Vibratory shear enhanced processing membrane filtration for the removal of natural organic matter from surface waters. J of Membrane Science **2006**, *269*, 1-14.

Atkinson, S. VSEP vibratory membrane filtration system treats hog manure. Membrane Technology **2005**, *1*, 10-11.

Jarusutthirak, C.; Mattaraj, S.; Jiraratananon, R. Influence of inorganic scalants and natural organic matter on nanofiltration membrane fouling. J of Membrane Science **2007**, *287*, 138-145.
